# Supplementary material for: Single Cell Analysis of Bistable Expression of Pathogenicity Island 1 and the Flagellar Regulon in Salmonella enterica
Source: Microorganisms. 2021 Jan 20;9(2):210. doi: 10.3390/microorganisms9020210 (PMC7909444; doi:10.3390/microorganisms9020210)
Supplement: Supplementary file 1 [file microorganisms-09-00210-s001.zip › Figure Supplementary/Figure S1.pdf]

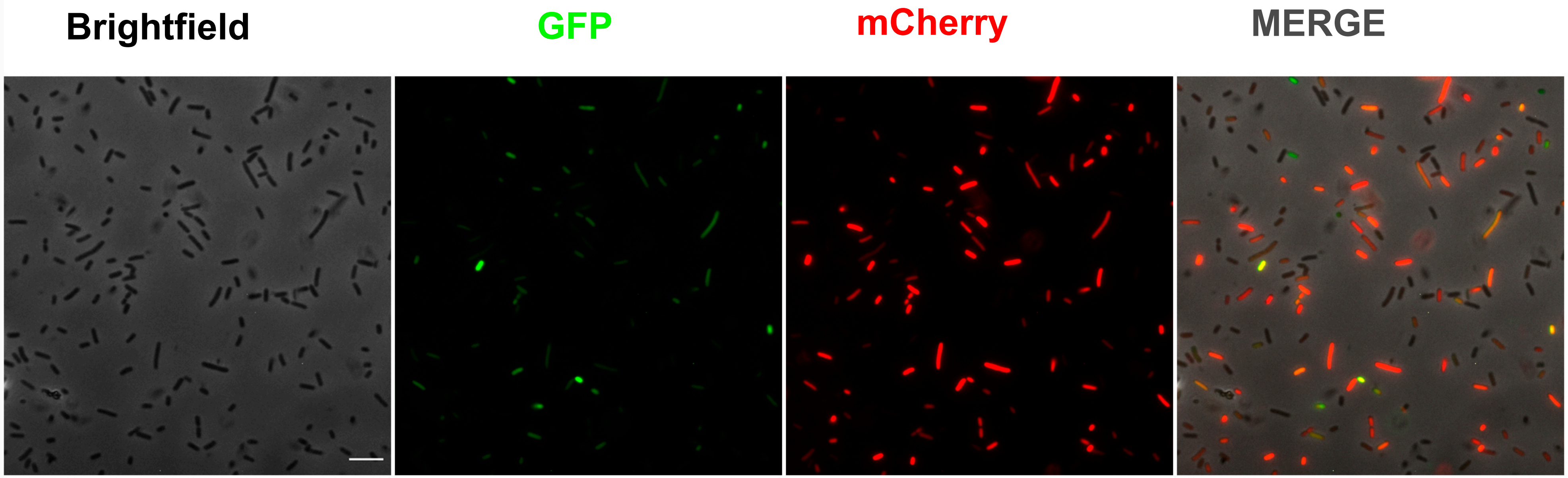

Figure S1. Fluorescence microscopy images of *S.* Typhimurium cells expressing SPI-1 (GFP) and/or flagelar (mCherry) regulon.
