# Supplementary material for: Single Cell Analysis of Bistable Expression of Pathogenicity Island 1 and the Flagellar Regulon in Salmonella enterica
Source: Microorganisms. 2021 Jan 20;9(2):210. doi: 10.3390/microorganisms9020210 (PMC7909444; doi:10.3390/microorganisms9020210)
Supplement: Supplementary file 1 [file microorganisms-09-00210-s001.zip › Figure Supplementary/Figure S2.pdf]

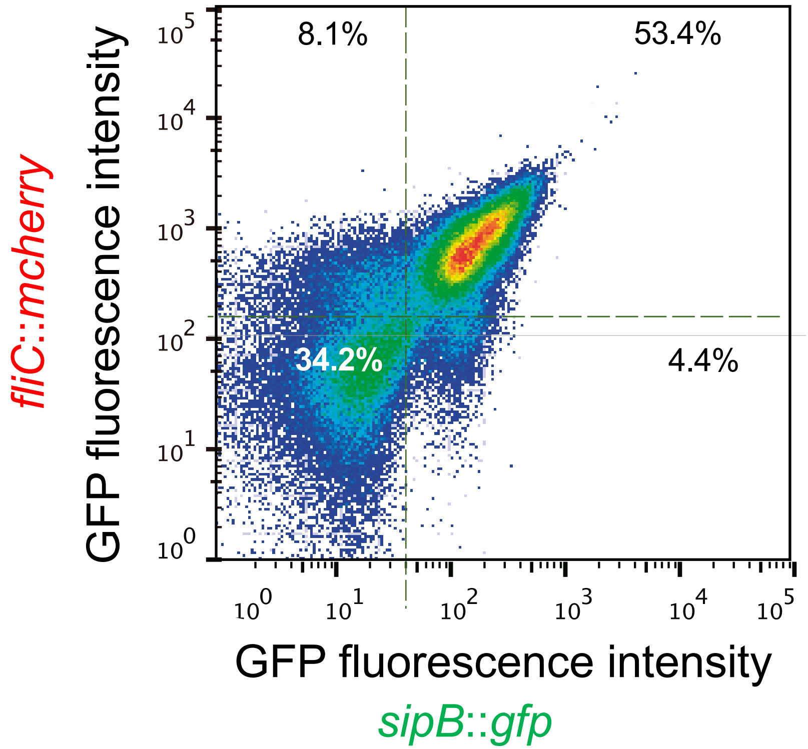

Figure S2. Single cell analysis of expression of SPI-1 (invasion, *sipB* gene) and flagellar (motility, *fliC* gene) genes at 37ºC in LB under aerobiosis.
